# Long COVID challenges in Brazil: an unfinished agenda for the Brazilian Unified National Health System

**DOI:** 10.1590/0102-311XEN008724

**Published:** 2024-02-19

**Authors:** Alberto Novaes Ramos

**Affiliations:** 1 Programa de Pós-graduação em Saúde Pública, Universidade Federal do Ceará, Fortaleza, Brasil.; 2 Departamento de Saúde Comunitária, Universidade Federal do Ceará, Fortaleza, Brasil.

The COVID-19 pandemic represented an unprecedented public health and humanitarian crisis in the history of humanity and, from a syndemic perspective, caused unequal and long-term direct and indirect effects on the population combined with political, economic, social, environmental, and individual issues ^1^ ^,^ ^2^ ^,^ ^3^.

From the first cases in 2019 to December 31, 2023, the cumulative number of global cases and deaths reported to the World Health Organization (WHO) was impressive, despite the number of underreported cases: almost 774 million people affected, of which, more than 38 million in Brazil (4.9%). In total, COVID-19 caused 7 million deaths including 708,000 in Brazil (10%) ^4^ ^,^ ^5^.

With the WHO declaring COVID-19 a global health emergency in February 2020, global efforts have focused on reducing morbidity, with measures to prevent the spread of SARS-CoV-2, vaccination to prevent infection and severe clinical forms, and reducing mortality ^2^ ^,^ ^6^. Brazil, despite the response of Brazilian Unified National Health System (SUS, acronym in Portuguese), has negative inter-federative articulation and coping strategies ^1^ ^,^ ^2^.

In addition the significant number of cases and deaths, COVID-19 is recognized as a chronic condition with high morbidity and mortality, despite being neglected by governments, researchers, health professionals, society in general, and people affected ^2^ ^,^ ^6^ ^,^ ^7^ ^,^ ^8^ ^,^ ^9^ ^,^ ^10^ ^,^ ^11^.

This clinical syndrome was initially called “long COVID” by patients with strong social media engagement and mobilization in advocacy strategies. The idea was to raise awareness and give visibility to an emerging public health problem ^3^ ^,^ ^8^ ^,^ ^9^ ^,^ ^11^ ^,^ ^12^. The WHO officially recognized it in August 2020 ^13^, and defined it as a “post-COVID-19 condition” in people with probable or confirmed SARS-CoV-2 infection, usually with persistent signs/symptoms three months after infection and lasting at least two months, and which are not explained by another diagnosis ^8^ ^,^ ^12^ ^,^ ^13^.

Other names are found in the literature, with specific definitions but limited consensus: “post-COVID syndrome” (National Institute for Health and Care Excellence/United Kingdom), “post-COVID-19 conditions” (Centers for Disease Control and Prevention/United States), “persistent symptoms or consequences of COVID-19”, and “post-acute sequelae of SARS CoV-2 infection” ^3^ ^,^ ^8^ ^,^ ^12^.

Despite its importance, long COVID remains underestimated ^7^ ^,^ ^12^ ^,^ ^13^ ^,^ ^14^ ^,^ ^15^ ^,^ ^16^. A case definition has not been established yet according to agreed objective criteria that would allow systematic diagnosis in clinical practice, which has led negligence, demobilization, reduced access to healthcare, disability, impairment, stigma, and death ^9^ ^,^ ^12^ ^,^ ^14^. This systematization must involve health professionals, researchers, people affected, caregivers/family members, funders, and health managers ^6^ ^,^ ^12^.

The way in which long COVID has been defined and measured over time has a direct impact on prevalence estimates, leading to high variability ^16^. Estimates of prevalence range from 10% to 70% up to 24 months after SARS-CoV-2 infection ^3^ ^,^ ^10^ ^,^ ^16^ ^,^ ^17^. Even 24 months after infection, neuropsychological symptoms have been frequently reported ^10^.

Based on a conservative estimate of 10%, the prevalence of long COVID would be close to 75 million people worldwide; including 4 million in Brazil. The estimated prevalence ranges from 10% to 30% in non-hospitalised cases, 50% to 70% in hospitalised cases, and 10% to 12% in vaccinated populations ^16^ ^,^ ^18^. A population-based study showed a 20.9% reduction in the prevalence of long COVID with COVID-19 vaccination among adults who had COVID-19 in the United States, and 15.7% in a group of 158 countries ^19^.

Long COVID can affect pediatric individuals of all ages, but it is more common in adult females, with type 2 diabetes mellitus or advanced age, with preexisting comorbidities, without a complete vaccination schedule for COVID-19, with low income, lower educational level, higher severity of the acute phase, and hospitalization in intensive care units (ICU) ^3^ ^,^ ^14^ ^,^ ^18^. However, more than 30% of people with long COVID may have no pre-existing conditions ^16^. Numerically, most cases of long COVID occur in non-hospitalized people with mild acute illness, which corresponds to most cases of COVID-19 in the world ^16^.

The prevalence of long COVID in women with acute infection and disease during pregnancy was similar to that in the general population ^20^. Among healthcare workers, being female and having been diagnosed with two or more SARS-CoV-2 infections were associated with long COVID ^21^. Healthcare workers with confirmed infection by Delta and Omicron variants of SARS-CoV-2 and who had received four doses of the COVID-19 vaccine prior to infection were less susceptible to long COVID ^10^ ^,^ ^18^ ^,^ ^21^.

From an intersectional perspective, individuals who are female, less educated, from a sexual or gender minority, and Hispanic or multiracial ethnicity are more susceptible to long COVID and activity limitations ^22^.

Reported clinical events include chronic fatigue syndrome (myalgic encephalomyelitis), dyspnea, impairment of physical and cognitive performance (loss of memory and concentration problems), headache, cardiovascular/thrombotic/cerebrovascular disease, type 2 diabetes mellitus, cough, hair loss, loss of smell and taste, and dysautonomia (postural orthostatic tachycardia syndrome), among others ^10^ ^,^ ^12^ ^,^ ^16^. These events can be mild or disabling, with potential reduction of health-related quality of life, frequent use of healthcare services, absenteeism, and indirect healthcare costs ^15^.

The first publication on long-term COVID-19 symptoms was written by researchers affected by long COVID (Patient-Led Research Collaborative) ^16^. Long COVID can affect various organs and systems of the human body, with a wide variety of reported signs/symptoms (more than 200 have been reported), that may persist or fluctuate, requiring multidisciplinary care ^2^ ^,^ ^12^ ^,^ ^16^ ^,^ ^22^.

Based on the challenges related to COVID-19, the CSP, in its 40 years to be celebrated in 2024, has contributed since 2020 with open access articles with critical reflection on the pandemic. More than 185 articles addressing different aspects of the pandemic have been published, the latest one included in this issue, two of which deal with the occurrence of long COVID: *Post-COVID-19 Syndrome among Hospitalized COVID-19 Patients: A Cohort Study Assessing Patients 6 and 12 Months after Hospital Discharge*, by Rocha et al. ^23^, and *Post-COVID-19 Syndrome: Persistent Symptoms, Functional Impact, Quality of Life, Return to Work, and Indirect Costs - A Prospective Case Study 12 Months after COVID-19 Infection*, by Ida et al. ^24^.

The study by Rocha et al. ^23^ was conducted in hospital units in the state of Mato Grosso, Brazil - with a high mortality rate due to COVID-19 -, and aimed to analyze long COVID among adults 6 and 12 months after hospital discharge due to COVID-19. It was the first study to evaluate this perspective in the state so it used an ambidirectional cohort designed at the end of 2021 and in early 2022. Data from 259 hospitalized people were collected from medical records and telephone contacts. The occurrence of persistent or new signs/symptoms was assessed, and their frequency was evaluated in the sociodemographic, economic, hospital admission and health status dimensions, recognizing potential limitations, such as the adopted definition of long COVID.

The prevalence of long COVID was estimated to be 88.4% at six months and 67.5% at 12 months, and was higher among people of an older age, those with lower per capita income, those without a job six months after discharge, those with systemic hypertension or type 2 diabetes mellitus, and with high severity during hospitalization in the acute phase. The process of increased vulnerability of these patients was also assessed ^23^.

Fatigue, dyspnea, arthralgia, memory loss, hair loss, and anxiety were the most common clinical events. The finding that less than half of the individuals studied had completed the vaccination schedule prior to hospitalization ^23^ needs be highlighted because of its importance for the prevention of long COVID, even at longer follow-up periods such as 24 months ^10^ ^,^ ^19^ ^,^ ^21^.

The study by Ida et al. ^24^ aimed to describe the clinical syndrome of long COVID, focusing on neurological symptoms and impact on cognitive, emotional, and motor functions; quality of life; and indirect costs due to loss of work productivity 12 months after acute infection in early 2021. All 58 patients analyzed in the study sought care for signs/symptoms of long COVID in a unit of the SARAH Network of Rehabilitation Hospitals in the state of Ceará, Brazil, and were contacted at the baseline and 12 months after infection.

About 67% were hospitalized, 60% in the intensive care in the acute phase, and the most common symptoms after 12 months were general fatigue, memory changes, dyspnea, anxiety, and arthralgia, with functional changes. The study showed impressive numbers, such as the estimated indirect costs nearly USD 130,000 (about USD 5,000 per person per year), with about 12,000 lost workdays - almost 1/3 of people did not return to work ^24^.

Taken together, these two articles highlight the importance of further studies on occurrence and impact of long COVID in the country with population representativeness due to its significant prevalence, and individual and collective effects. The high level of uncertainty associate with knowledge gaps ^2^ ^,^ ^8^ ^,^ ^12^ needs to be addressed to support the decision-making process. In addition to the heterogeneity of terminologies and classifications used in the long COVID, the limited standardization of methods is a result of the variability of primary outcomes and the imprecise diagnostic and inclusion criteria adopted over time ^3^ ^,^ ^8^ ^,^ ^12^ ^,^ ^13^ ^,^ ^17^.

These issues are still considered challenges, as are the definition of risk factors, diagnostic criteria, and effective treatments ^3^ ^,^ ^12^ ^,^ ^16^. Various mechanisms underlying the pathogenesis have been suggested to explain the complexity of long COVID ^10^ ^,^ ^12^ ^,^ ^16^ ^,^ ^17^. Studies integrating the underlying mechanisms with a focus on biomarkers and the impact of vaccination on long-term outcomes are also strategic ^10^ ^,^ ^19^.

There are persistent barriers in health services for diagnosis and longitudinal care of people with long COVID, particularly those with a higher susceptibility to infection. Studies on health needs and access to and quality of health services should be prioritized, as well as assessment and monitoring of economic impact ^6^ ^,^ ^14^ ^,^ ^22^ ^,^ ^25^. The time of diagnosis of acute SARS-CoV-2 infection is an opportunity for qualified counseling due to the potential risk of long COVID, with patient empowerment focusing on empathic listening, awareness, responsibility, and health surveillance. For example, older adults may have underreported long COVID symptoms due to mistaken association with typical comorbidities of this period. Therefore, qualitative perspectives of individual and collective effects and impacts on health systems beeds to be included into research agendas.

In May 2023, the WHO declared the end of the global health emergency of COVID-19 with an increase in vaccination coverage and a reduction in the number of acute cases and deaths. Given the wide spread of SARS-CoV-2 infection around the world, particularly in countries with high rates of the disease, such as Brazil, the burden of long COVID is likely to remain relevant ^8^ ^,^ ^12^ ^,^ ^23^ ^,^ ^24^, challeging the sustainability of research agendas ^6^. There isare concerns about demobilization of various stakeholders and increased invisibility of long COVID, particularly in contexts of high social vulnerability ^3^ ^,^ ^11^ ^,^ ^12^.

Meanwhile, millions of people affected by long COVID around the world articulate support for timely and qualified access to diagnosis and longitudinal care ^3^, facing psychosocial and physical effects to overcome the associated stigma ^22^.

In Brazil, strategic policies are needed to expand access to the care network and actions of the immunization program under the SUS, especially in primary health care, and to strengthen the education and social security networks, with a focus on reducing serious socioeconomic inequalities ^6^ ^,^ ^7^ ^,^ ^11^ ^,^ ^12^ ^,^ ^25^ ^,^ ^26^. Adequate financing to the health sector for prevention, diagnosis, and treatment based on comprehensive care, in combination with other intersectorial public policies, should be a goal ^2^ ^,^ ^6^ ^,^ ^7^. It means democratically overcoming the economic model based on austerity in order to reduce social inequalities and expand the financing and management capacity of the SUS ^1^ ^,^ ^2^ ^,^ ^7^ ^,^ ^25^ ^,^ ^26^.

Therefore, it involves is a number of essential strategies for a broad national response to long COVID, which has emerged as a critical public health problem in the SUS. It must integrate support to overcoming direct and indirect negative effects on people affected, their families and communities, and society as a whole.
